# Synthesis and Characterization of Stiff, Self-Crosslinked Thermoresponsive DMAA Hydrogels

**DOI:** 10.3390/polym12061401

**Published:** 2020-06-22

**Authors:** Juan Carlos Rueda, Fátima Santillán, Hartmut Komber, Brigitte Voit

**Affiliations:** 1 Polymer Laboratory, Physics Section, Research Department (DGI), Pontifical Catholic University of Peru (PUCP), 15088 San Miguel, Peru; fsantillane@pucp.edu.pe; 2 Leibniz Institute of Polymer Research Dresden, Hohe Strasse 6, 01069 Dresden, Germany; voit@ipfdd.de

**Keywords:** DMAA hydrogel, thermosensitivity, self-crosslinking, macromondomer, polyoxazoline

## Abstract

Stiff thermosensitive hydrogels (HG) were synthesized by self-crosslinking free radical polymerization of *N,N*-dimethylacrylamide (DMAA) and N-isopropylacrylamide (NIPAAm), adjusting the degree of swelling by carboxylate-containing sodium acrylate (NaAc) or a 2-oxazoline macromonomer (MM). The formation of hydrogels was possible due to the self-crosslinking property of DMAA when polymerized with peroxodisulfate initiator type. The MM was synthetized by the ring-opening cationic polymerization of 2-methyl-2-oxazoline (MeOxa) and methyl-3-(oxazol-2-yl)-propionate (EsterOxa), and contained a polymerizable styryl endgroup. After ester hydrolysis of EsterOxa units, a carboxylate-containing MM was obtained. The structure of the hydrogels was confirmed by ^1^H high-resolution (HR)-MAS NMR spectroscopy. Suitable conditions and compositions of the comonomers have been found, which allowed efficient self-crosslinking as well as a thermoresponsive swelling in water. Incorporation of both the polar comonomer and the macromonomer, in small amounts furthermore allowed the adjustment of the degree of swelling. However, the macromonomer was better suited to retain the thermoresponsive behavior of the poly (NIPAAm) due to a phase separation of the tangling polyoxazoline side chains. Thermogravimetric analysis determined that the hydrogels were stable up to ~ 350 °C, and dynamic mechanical analysis characterized a viscoelastic behavior of the hydrogels, properties that are required, for example, for possible use as an actuator material.

## 1. Introduction

In recent years, the field of smart hydrogels has generated great technological interest due to the variety of applications in which they can be used, since these materials are able to respond in a reversible way to external stimuli such as pH value, temperature, light, etc., [1,2]. Among the main applications of these polymeric materials are controlled release of drugs [3,4,5,6], immobilization of enzymes and proteins [7,8], and as active materials in sensors and actuators [9], among others.

Smart hydrogels can be synthesized based on one or several monomers, according to the type of properties that one wants to assign to the material. N-isopropylacrylamide (NIPAAm) is commonly used when obtaining a temperature-sensitive hydrogel with a transition temperature close to human body temperature is desired. PolyNIPAAm presents a conformational transition phenomenon (Lower Critical Solution Temperature, LCST) in water, where the polymeric segments start to interact with themselves stronger than with water, when the temperature equals or exceeds 32 °C [10].

Other factors that determine both the properties of these hydrogels, and therefore, their applicability are the functional groups of the monomers and the degree of crosslinking. Geckeler et al. reported the synthesis of hydrogels based on NIPAAm copolymerized with sodium acrylate [11], demonstrating that by using hydrophilic monomers such as sodium acrylate, an improvement in water absorption capacities was generated and additionally, pH sensitivity was obtained.

On the other hand, Needles and Whitfield [12] discovered the self-crosslinking property of *N,N*-dimethylacrylamide (DMAA), when this monomer was polymerized in water via free radicals using peroxodisulfate-type initiators. Using this property, Cipriano et al. [13] produced *N,N*-dimethylacrylamide hydrogels without the use of conventional crosslinking agents via radical polymerization initiated by potassium peroxodisulfate. The hydrogels obtained showed a great water absorption capacity (superabsorbent) and excellent mechanical properties (elongation up to 1350%). This last property is probably due to the specific self-crosslinking mechanism that is assumed to produce a homogeneous distribution of the crosslinking points in the hydrogel [13].

Recently, Sun et al., also using the self-crosslink capacity of dimethylacrylamide, obtained dimethylacrylamide and sodium acrylate hydrogels by photoinitiation with camphorquinone and diphenyliodonium hexafluorophosphate, obtaining a hydrogel with excellent mechanical properties and high water absorption [14]. Thus, the self-crosslinking ability of DMAA allows for a very simple gel formulation without any influence of chemically different crosslinking moieties and leads to a favorable, homogeneous gel structure with high water absorption ability.

The aim of the present study was to combine the self-crosslinking behavior of DMAA, leading also to hydrogels with a high water absorption capacity, with the thermoresponsivity of polyNIPAAm. The challenge was to adjust the composition in a way that both features are retained, sufficient crosslinking achieves tough hydrogels, as well as a significant temperature-induced change in the degree of swelling from the thermoresponsive comonomer. In order to compensate for the reduced amount of DMAA, additional polar carboxylate units should be incorporated.

In order to achieve a strong thermoresponsive behavior in a PNIPAAm hydrogel as well keeping the transition temperature close to the human body temperature, random distribution of additional polar monomer units is unfavorable. The formation of interpenetrating networks [15] or of graft copolymer gels [16,17,18], however, are very suitable methods to achieve the desired phase-separating structures needed to retain the PNIPAAm behavior as well as obtaining gels with high mechanical strength. A versatile approach towards graft copolymer gels is the use of macromonomers, carrying e.g., polar or pH sensitive function, framing the network structure by the thermoresponsive monomer and a crosslinker. This allows the achieving of a gel framework with tangling polymer chains from the macromonomer that can phase-separate, allowing the formation of a relatively undisturbed PNIPAAm phase. Specifically, poly (2-oxazoline) macromonomers are well suited for this. The synthesis of poly (2-oxazoline)s by ring-opening cationic polymerization proceeds in a ‘living’ form without termination or chain transfer reactions, thus, the molecular weight as well as the end functionality of these polymers can be well controlled, allowing the introduction of polymerizing units [19]. In addition, oxazoline monomers can be fine-tuned to carry pH as well as temperature sensitivity. Thus, recently, we reported on bisensitive graft copolymers made from a thermoresponsive styryl-terminated poly (2-cyclopropyl-2-oxazoline) macromonomer and pH responsive sodium acrylate [20]. Previously, pH as well as thermoresponsive graft copolymers were achieved using N-isopropylacrylamide and a pH responsive macromonomer consisting of a copolymer of 2-carboxyethyl- and 2-methyl-2-oxazoline, crosslinked with a bisacrylamide [21].

In the present article, we report on the synthesis of temperature sensitive PNIPAAm hydrogels, using, in addition, DMAA simultaneously as a monomer favoring water absorption and as a self-crosslinking agent. Degree of swelling was adjusted by incorporation of small amounts of carboxylate-containing 2-oxazoline macromonomer (MM) and in comparison, by random incorporation of sodium acrylate (NaAc). Thus, the aim was to combine the features of a thermoresponsive graft copolymer hydrogel that had a strong phase transition at favorable temperature and high stiffness, with the homogeneous crosslinking achieved through the DMAA/potassium peroxodisulfate system.

## 2. Materials and Methods

### 2.1. Materials

2-Methyl-2-oxazoline (MeOxa) and methyl-3-(oxazol-2-yl)-propionate (EsterOxa) were synthesized by methods described in the literature [22,23,24]. Acetonitrile (Aldrich), *N,N,N’,N’*-tetramethylethylenediamine (TEMED, Aldrich, St. Louis, MO, USA), MeOxa and EsterOxa were dried by distillation over calcium hydride. Chloromethylstyrene (CMS) (Aldrich, mixture of *meta* (70 mol%) and *para* isomers (30 mol%)) was purified by fractional distillation before use. *N*-Isopropylacrylamide (NIPAAm) (Aldrich) was recrystallized from absolute ethanol. *N,N*-Dimethylacrylamide (DMAA), sodium acrylate (NaAc) and potassium peroxodisulfate (KPS) (Merck) and further reagents and solvents were used as received.

### 2.2. Methods

The SEC analysis of the MM was carried out on an Agilent Technologies LC 1100 apparatus (Santa Barbara, CA, USA) equipped with a PL Oligopore column and RI detector. The sample was eluted with dimethylacetamide containing 2 vol % water and 3 g/L LiCl. Polyvinylpyridine standards were used for molecular weight calibration. The NMR measurements were carried out on a Bruker Advance III 500 spectrometer (Billerica, MA, USA) operating at 500.13 MHz for ^1^H. For high resolution-magic angle spinning (HR-MAS) measurements, the PTFE insert of the ZrO_2_ rotor (4 mm outer diameter, 50 μL insert volume) was filled with small pieces of dry hydrogel (~2 mg). After addition of D_2_O and about 30 min swelling time, the sample was measured with a HR-MAS probe (Bruker, Billerica, MA, USA) at υ_r_ = 4000 Hz. The ^1^H NMR spectra were referenced on the HDO signal (4.72 ppm). The rheological measurements were made with the dynamic mechanical analyzer ARES-G2 (TA Instruments, New Castle, DE, USA) in the angular speed range of 0.1 to 10 rad/s, with an oscillation strain of 0.01 and parallel plate geometry at a temperature of 22 °C, with an axial force of 0.3 N and a deformation control of 0.5%. For the rheological measurements, the hydrogels were immersed in distilled water for 24 h to reach the equilibrium. After that, the swollen hydrogels were removed from water and tested. The pH values were adjusted with 0.1 N NaOH and 0.1 N HCl solution and measured with a pH meter and conductometer Mettler Toledo Seven Multi (Columbus, OH, USA). TGA thermograms were obtained with a Netzsch STA 449 F3 Jupiter simultaneous thermal analyzer (Selb, Germany) in a range of 30–600 °C at a heating rate of 10 K/min under a nitrogen atmosphere (50 mL/min).

### 2.3. Procedures

#### 2.3.1. Synthesis of 2-Oxazoline Macromonomer (MM)

The MM was synthesized according to the method described in the literature [20]. In total, 6.89 g (81.0 mmol) MeOxa, 4.24 g (27.0 mmol) EsterOxa, 1.62 g (10.88 mmol) NaI and 0.828 g (5.4 mmol) of CMS were reacted in 22 mL of acetonitrile to the macromonomer in 96% yield. Based on the ^1^H NMR spectrum, the degree of polymerization (DP) was determined to be 24, and a molar content of 78 mol% MeOxa and 22 mol% EsterOxa was calculated. The calculations are based on a molecular weight of 2550 g/mol.

^1^H NMR (CD_3_OD) δ: 7.5–7.1 (H_arom_), 6.74 (=CH–), 5.80 and 5.25 (=CH_2_), 4.65 (Ar–CH_2_–), 3.68 (OCH_3_), 3.65–3.4 (N–CH_2_–CH_2_–), 2.95–2.55 (NC(O)CH_2_CH_2_CO), 2.2–2.0 ppm (NC(O)CH_3_).

#### 2.3.2. Synthesis of Hydrogels (HG)

The hydrogels were synthesized via radical polymerization using the copolymerization systems: a) DMAA-NIPAAm-NaAc (HG-1 to -4), b) DMAA-NIPAAm (HG-5) and c) DMAA-NIPAAm-MM (HG-6 to -9). The hydrogel synthesis was carried out based on the method reported by Cipriano et al. [13]. Here, the self-crosslinking capacity presented by DMAA is used in the presence of peroxodisulfate-type radical initiators. Typical procedure (HG-6): A mixture of 1.005 g (10.14 mmol) DMAA, 0.058 g (0.023 mmol) MM, and 1.752 g (15.48 mmol) NIPAAm in water was bubbled for 45 min with nitrogen. After this time, 0.028 g (0.10 mmol) of KPS were added and the solution was bubbled with nitrogen for a further 15 min. Finally, four drops of TEMED were added and the final mixture was placed in a glass mold left at 5 °C until gelation. Then, it was left at 25 °C for 12 h, before beginning the purification of the hydrogel. The hydrogel was purified by repeated washing with distilled water, dried in a vacuum at 40 °C, and stored in a desiccator. The experimental details and results are summarized in Table 1 and Table 2.

HG-1-HG-5: HR-MAS ^1^H NMR (D_2_O) δ: 3.89 (CH(CH_3_)_2_, NIPAAm), 3.2–2.25 (CH and N(CH_3_)_2_, DMAA), 2.25–1.25 (CH_2_, DMAA; CH and CH_2_, NIPAAm), 1.15 ppm (CH_3_, NIPAAm). Signals of NaAc comonomer could not be identified due to its low content.

HG-6-HG-9: HR-MAS ^1^H NMR (D_2_O) δ Type Equation There: 3.89 (CH(CH_3_)_2_, NIPAAm), 3.69 (OCH_3_, MM), 3.65–3.4 (N-CH_2_-CH_2_-, MM), 3.2–2.25 (CH and N(CH_3_)_2_, DMAA), 2.25–1.25 (CH_2_, DMAA; CH and CH_2_, NIPAAm; NC(O)CH_2_CH_2_CO and NC(O)CH_3_, MM), 1.15 ppm (CH_3_, NIPAAm).

#### 2.3.3. Hydrolysis of MM-Based Hydrogels

To remove the methyl ester group of the EsterOxa comonomer in the MM, a hydrolysis under basic conditions was carried out. Typical procedure: a portion of 4 g of HG-6, swollen in water, was immersed in 15 mL of 0.1 N NaOH at 35 °C for 48 h. After this time, the hydrolyzed hydrogel (HG-H) was purified by washing with distilled water until neutrality. Finally, the hydrogel was dried in a vacuum at 40 °C and stored in a desiccator. HG-6H: HR-MAS ^1^H NMR (D_2_O): The spectrum corresponds to the spectrum of HG-6, except that the signal at 3.69 ppm (OCH_3_) disappeared due to the hydrolysis.

#### 2.3.4. Water Absorption Degree (Q)

The water absorption degree Q of the hydrogels was calculated from the weight of a swollen piece of hydrogel (W_s_) and of the same piece after drying (W_d_), according Equation (1). For freshly prepared gels, the W_s_ value was obtained directly on the swollen gel after synthesis and purification (after removing surface water by gently swept with tissue paper), and then, the gel was dried in a vacuum at 40 °C until it reached a constant weight (W_d_). However, predominantly, the thoroughly dried and stored hydrogel was reswollen in deionized water (pH = 5.7) at 20 °C for 24 h and weighed after removing surface water by gently sweeping with tissue paper to obtain W_s_. Then, the hydrogel was dried again in a vacuum at 40 °C until it reached a constant weight (W_d_).

Q = (W_s_ − W_d_)/W_d_ (1)

#### 2.3.5. Sensitivity to Temperature

The sensitivity to temperature was expressed, in macroscopic terms, as a contraction or expansion of the hydrogel volume. It was considered that the weight of the swollen hydrogel was always proportional to its volume, therefore, instead of measuring the volume, the weight was measured. To avoid errors due to surface water, all swollen HG pieces were gently swept with tissue paper before weighing. Generally, about 3 g of the hydrogel was used in the swelling experiments. The sensitivity to temperature was concluded from the remaining water (%W_r_) of the swollen hydrogel at different temperatures. The reference weight (W_initial_) was determined for a portion of hydrogel immersed in water at 18 °C for 24 h. Then, this portion was immersed in water at different temperatures up to 70 °C for 1 h and weighed again (W_T_).

%W_r,T_ = (W_T_/W_initial_) × 100 (2)

#### 2.3.6. Water Absorption and Desorption Kinetics

For the water absorption kinetics test, the Q value (Equation (1)) of a HG piece was determined first after 30 min immersion time in water at 25 °C. This process of water uptake and weighing was continued on this sample up to 720 min swelling time. For the water desorption kinetic test, the %W_r,t_ value (Equation (3)) of a HG piece, initially swollen at 25 °C, in water at 60 °C, was followed. The reference weight (W_t=0_) was determined for a portion of hydrogel immersed in water at 25 °C. Then, this piece was immersed in water at a temperature of 60 °C for a period of t = 30 min and W_t=30_ was determined. The same procedure was applied for several deswelling times up to 420 min.

%W_r,t=x_ = (W_t=x_/W_t=0_) × 100 (3)

## 3. Results and Discussion

### 3.1. Synthesis of Poly (2-Oxazoline) Macromonomer

**Scheme 1:**
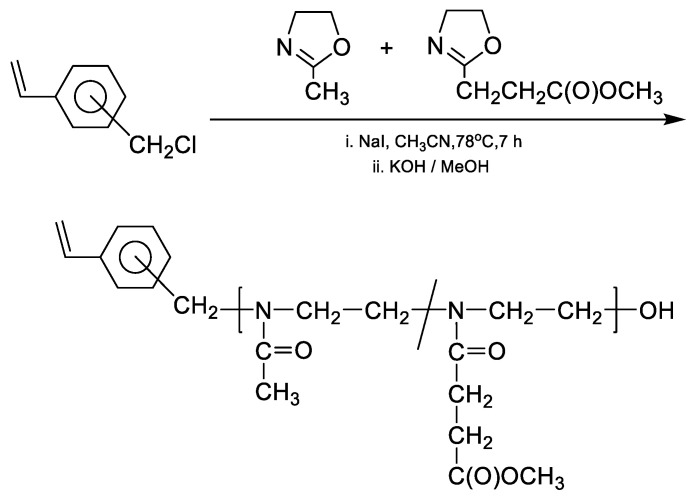
Synthesis of 2-oxazoline macromonomer (MM).

The MM was synthesized by cationic ring-opening polymerization of 2-methyl-2-oxazoline (MeOxa) and methyl-3-(oxazol-2-yl)-propionate (EsterOxa) initiated by chloromethylstyrene (CMS) (Scheme 1). Sodium iodide was added to coinitiate the reaction by halogen exchange. To avoid polymerization of the styryl starting group, a low concentration of CMS (0.17 M), a relatively low reaction temperature of 78 °C, and a short reaction time of 7 h was used [21]. The polymerization was terminated with methanolic KOH introducing an OH endgroup. The structure of the MM was confirmed by ^1^H NMR spectroscopy. The content of each monomer in the macromonomer was determined from the integrals of the methyl signal of MeOxa (2.2–2.0 ppm) and the C(O)CH_2_CH_2_C(O) methylene groups of EsterOxa (2.95–2.55 ppm). Considering the number of protons contributing to each integral, a molar content of 78 and 22% of MeOxa and EsterOxa, respectively, was determined in good agreement with the theoretical values (75 and 25%, respectively). Relating the sum of the intensity of one MeOxa and one EsterOxa proton to the intensity of one styryl endgroup proton results in a degree of polymerization (DP) of 24 units (theoretical value: 20), providing about five ester groups in one MM chain. M_n,NMR_ (2550 ± 150 g/mol) is lower than the M_n,SEC_ value (3900 g/mol), whereby the SEC value is based on calibration with PVP standards. However, the low polydispersity (Ð = 1.10) confirms the living character of the polymerization. Bouten et al. found for the methyl p-toluenesulfonate-initiated copolymerization of MeOxa and EsterOxa at 140 °C under microwave irradiation, a preferred incorporation of MeOxa in the copolymer due to its greater nucleophilicity [24]. This suggests that also for the MM, a comonomer gradient could occur with the EsterOxa units closer to the chain end.

### 3.2. Synthesis of Hydrogels (HG) and Hydrolysed Hydrogels (HG-H)

**Scheme 2:**
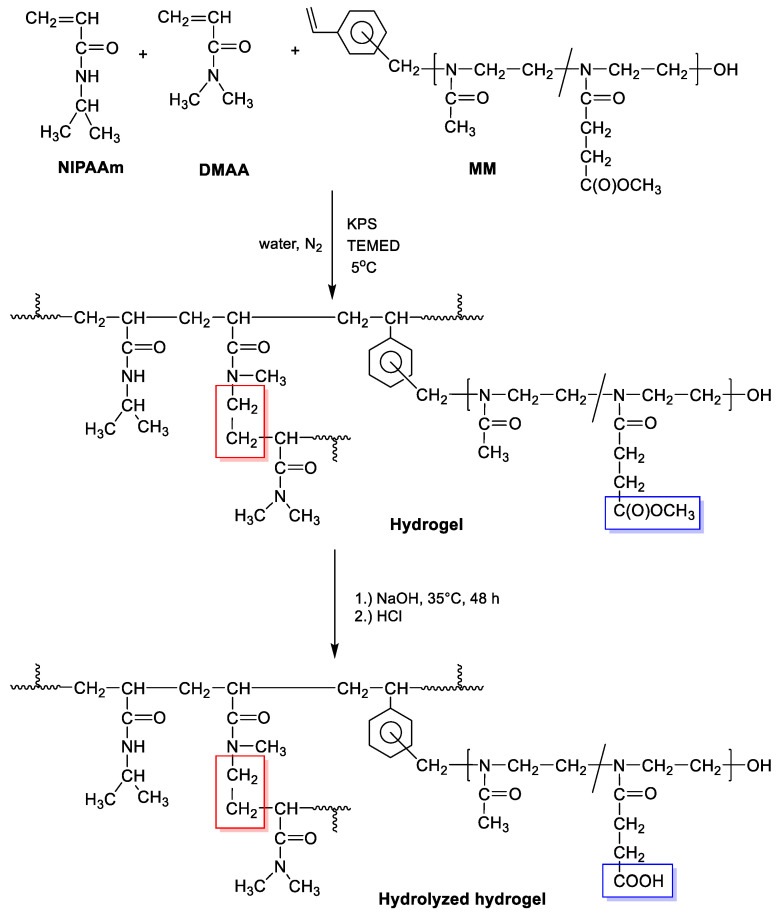
Synthesis of hydrogels HG-6H to -9H based on DMAA, NIPAAm and MM. The red box marks a branching/crosslinking point. MM has been replaced by sodium acrylate for gels HG-1 to -4. HG-5 was prepared only by NIPAAm and DMAA.

The hydrogels were obtained by free radical polymerization in water at 5 °C, of DMAA, NIPAAm and MM (or NaAc)), using potassium peroxodisulfate as an initiator and TEMED as a catalyst (Scheme 2). DMAA acts as a monomer but also as a crosslinking agent. In the first set of experiments (Table 1), the DMAA content varied between 82 and 19 mol% (HG-1–HG-4) in order to determine the limit which allows both thermoresponsive behavior as well as a sufficient crosslinking. In addition, the effect of a small amount sodium acrylate (below 1.66 mol%) on the degree of swelling was evaluated. HG-5 was prepared as a reference hydrogel of DMAA and NIPAAm without a pH sensitive comonomer. The gelation occurred rapidly within 10 min and resulted in transparent hydrogels, with the exception of HG-4, with the lowest DMAA content of 19 mol%. Thus, as described before, in addition to regular radical copolymerization, a self-crosslinking reaction efficiently took place. As a probable scenario, the formation of methylene radicals by attack of persulfate radical on a methyl group of DMAA is proposed [12,13]. These additional radical sites can initiate graft chains, and finally, result in the crosslinked hydrogel structure. For the second series (Table 2), a DMAA content of about 39 mol% was selected, since this allowed sufficient crosslinking but also a high content of thermoresponsive NIPAAm. However, in this series, the NaAc was replaced by the above-synthesized MM (Scheme 2).

**Scheme 3:**
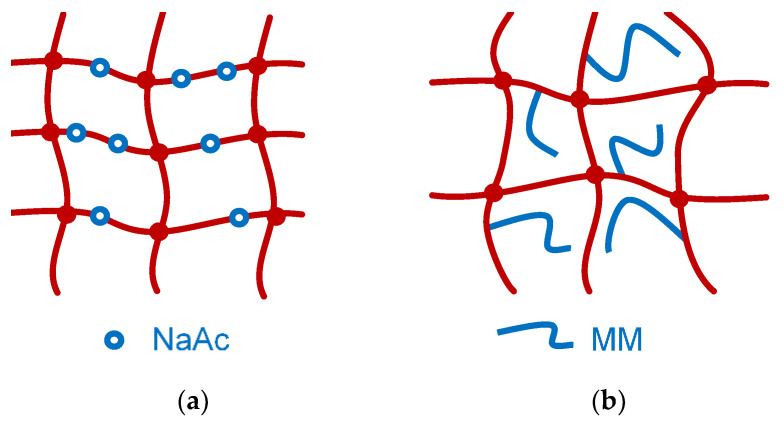
Sketch of: (**a**) hydrogels containing NaAc comonomers in the network; (**b**) hydrogels with macromonomer in the network.

The amount of the polyoxazoline macromonomer was varied from 0.09 to 0.52 mol%. Again, crosslinked and transparent hydrogels were achieved in all cases. Please note that compared to the copolymer networks obtained with NaAc, in the case of MM, so called graft copolymer networks with tangling MM chains are obtained, as indicated in Scheme 3. The ester groups of the MM-based hydrogels HG-6 to -9 were further converted to sodium salt form of carboxylic acid (HG-6H to -9H) by hydrolysis under mild basic conditions (0.1 N NaOH, 35 °C, 48 h). This allowed for selective hydrolyzation of the ester groups of MM and avoidance of possible hydrolysis of the amide groups [21]. As seen in Table 2, since about five COOH groups are introduced with one MM molecule, the COOH content varied between 0.45 to 2.6 mol%.

### 3.3. Characterization of Hydrogels

#### 3.3.1. HR-MAS ^1^H NMR Spectroscopy

**Figure 1:**
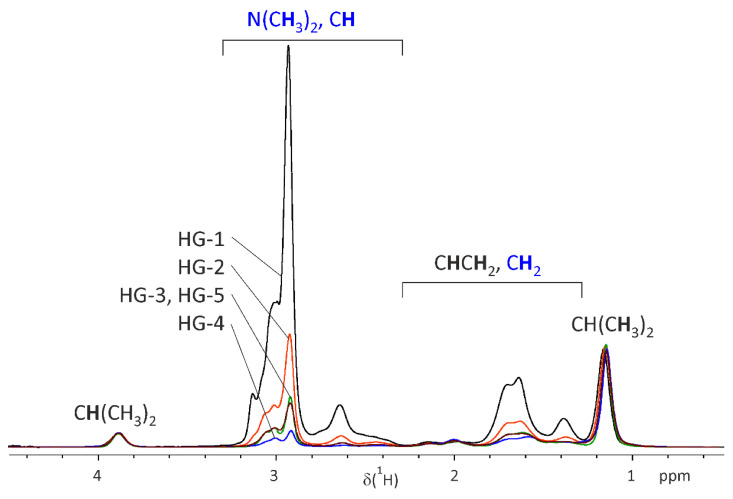
HR-MAS ^1^H spectra of HG-1 to -5 swollen in D_2_O. The spectra of HG-3 and HG-5 are almost identical. The signal assignments refer to DMAA (blue) and NIPAAm (black) comonomer.

**Figure 2:**
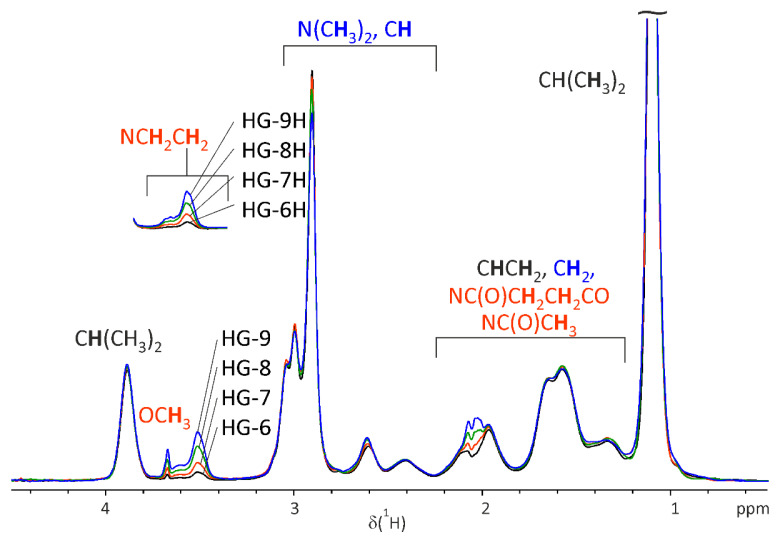
HR-MAS ^1^H spectra of HG-6 to -9 swollen in D_2_O. The inset shows the 3.4–3.8 ppm region for the hydrolyzed hydrogels HG-6H to -9H. The signal assignments refer to DMAA (blue), NIPAAm (black) and MM (red) comonomer.

The characterization of the hydrogels swollen in D_2_O by HR-MAS ^1^H NMR spectroscopy allowed for corroboration of the expected structure of the hydrogels (Figure 1 and Figure 2). The DMAA-NIPAAm-NaAc (HG-1–HG-4) and DMAA-NIPAAm (HG-5) hydrogels result in similar ^1^H NMR spectra at a qualitative level. A quantitative analysis based on the signal integrals of the 4.05–3.7 ppm region (one proton of NIPAAm comonomer) and 3.2–2.25 ppm region (seven protons of DMAA comonomer) allowed the determination of the molar content of each monomer in the hydrogel. The low content of NaAc comonomer was neglected in these calculations. The obtained values are in good agreement with the theoretical ones (Table 1). Figure 2 depicts the HR-MAS ^1^H NMR spectra of the hydrogels HG-6–HG-9. As expected, the spectra are very similar to the spectrum of HG-5 but additional signals of the MM comonomer occur. In the 3.8–3.4 ppm region, the methyl ester group appears as a singlet at 3.69 ppm and the NCH_2_CH_2_ protons appear as a signal group. Additionally, the C(O)CH_2_CH_2_C(O) signal group (~2.7 ppm) and the NC(O)CH_3_ signal group (~2.15 ppm) can be identified but overlap with DMAA and NIPAAm signals. After hydrolysis, the methyl ester signal disappears for HG-6H to HG-9H (Figure 2, inset), indicating complete conversion. The remaining NCH_2_CH_2_ signals in the 3.8–3.4 ppm region represent n-times four protons of the MM comonomer. For calculation of the comonomer ratio (Table 2), integrals of NIPAAm and DMAA protons were used. The DMAA integral has to be reduced by the intensity of the C(O)CH_2_CH_2_C(O) protons [0.22 × I (3.8–3.4 ppm)] of MM. Despite the calculations being error prone, the content of MM in the hydrogels is lower than expected from the feed ratio (Table 2). Probably, this is caused by the lower accessibility of the polymerizable styryl group in the coiled MM compared to the low molecular-weight DMAA and NIPAAm comonomers enhanced by reduced diffusivity due to fast gelation.

#### 3.3.2. Water Absorption and Desorption

**Figure 3:**
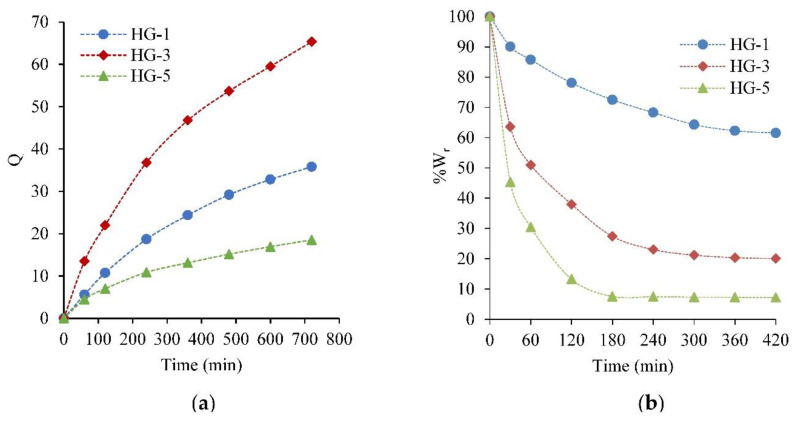
(**a**) Water absorption kinetics (expressed as water absorption degree) at 25 °C, (**b**) water desorption kinetics (expressed as percent of remaining water) at 60 °C for hydrogels HG-1, -3 and -5.

The water absorption degree Q was determined for all hydrogels at 20 °C and pH 5.7 (Table 1 and Table 2). Please note that we report two different Q values, one directly determined after the preparation and purification of the gel, and one which was obtained after thorough drying of the gel, storage, and subsequent reswelling. For DMAA self-crosslinking gels, a very high water absorption was found by Cipriano et al. [13] and Sun et al. [14]. Since the hydrophilicity of NIPAAm is lower than for DMAA, it was very important to optimize the composition of the gels. On the one hand, we wanted to achieve efficient self-crosslinking through DMAA units, but also strong temperature-induced volume change introduced by the NIPAAm units. Thus, we introduced, in addition, a small amount of polar comonomers which should allow compensation for the lower swelling degree of PNIPAAm compared to PDMAA and to achieve a strong volume change. In the first series (HG-1–HG-4), sodium acrylate was introduced in a small amount of 1.0 to 1.7 mol% and at the same time, the content of DMAA and NIPAAm was varied. Looking at the result, one has to keep the following aspect in mind: with higher DMAA content, a higher crosslinking density is observed, which dominates the swelling behavior. Finally, the highest Q value for the freshly prepared gels with 83 was found for HG-3, with about 39.3 mol% DMAA, 59.5 mol% of NIPAAM and 1.2 mol% NaAc, which was three times higher than the reference gel HG-5 (Q = 28) with a similar DMAA/NIPAAm composition, but without NaAc. Thus, it can be confirmed that the strategy to introduce a polar, carboxylate containing comonomer even as low as 1 mol% strongly enhanced the swelling capacity of the gel. HG-4 with the lowest content of crosslinking DMAA shows low mechanical stability and disintegrates in the swelling experiment. The time-dependency of swelling determined for HG-1, HG-3 and HG-5 at 25 °C shows the same tendency as HG-3, having the highest water absorption capacity (Figure 3a). The desorption kinetics tests were carried out at 60 °C (Figure 3b). At this temperature, one could expect an effect of the temperature-sensitive polyNIPAAm segments resulting in increasing hydrophobicity above the transition temperature. In fact, the observed desorption kinetics and the final %W_r_ values after 420 min reveal two influences. First, the lower NIPAAm content of HG-1 compared to HG-3 results in a lower water desorption rate. For HG-1, the polyNIPAAm content is lower, the transition temperature is shifted above 60 °C and only a low shrinking occurs. Second, a low content of NaAc reduces the hydrophobic effect of the NIPAAm comonomers also at higher temperatures, as can be concluded from the desorption behavior of HG-3 and HG-5.

**Figure 4:**
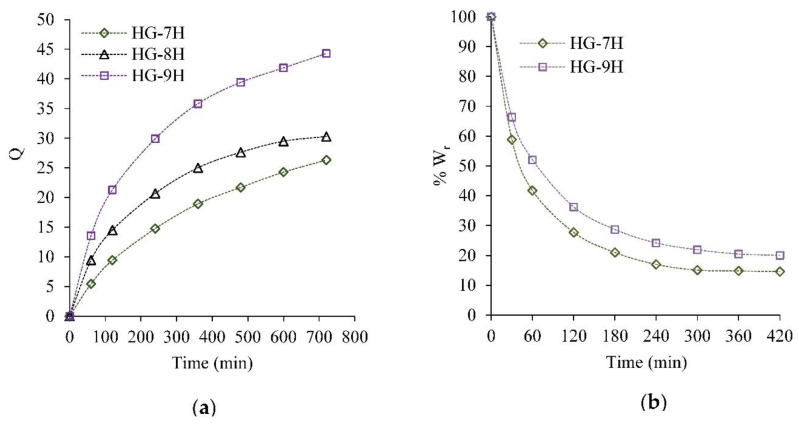
(**a**) Water absorption kinetics (expressed as water absorption degree) at 25 °C for HG-7H, -8H and -9H, (**b**) water desorption kinetics (expressed as percent of remaining water) at 60 °C for hydrogels HG-7H and HG-9H.

The HG-6 to -9 series and the corresponding hydrolyzed hydrogels have a very similar DMAA/NIPAAm ratio but slightly increased MM content from 0.09 to 0.52 mol%, which corresponds to a COOH content between 0.45 to 2.6 mol% after hydrolysis. The Q values of the hydrogels before hydrolysis show no dependency on the MM content (Table 2) and are very similar to the DMAA-NIPAAm hydrogel HG-5 of similar composition, but without MM comonomer. However, hydrolysis of the methyl ester groups to carboxylate groups results in a clear dependency of Q from the MM content, especially in the freshly prepared gels. Even for HG-6H with only 0.09 mol% MM, the Q value increases from 25 to 48 (Table 2). For HG-9H with 0.52 mol% MM (2.6 mol% COOH groups), the Q value increases still further to 93, exceeding the Q value of HG-3. The water absorption kinetics at 20 °C depicted in Figure 4a for HG-7H to HG-9H corroborate the faster swelling and higher swelling degree for HG-9H with the highest MM content. The desorption kinetics at 60 °C (Figure 4b) carried out for HG-7 and HG-9 are very similar because both hydrogels have a comparable NIPAAm content determining the transition to the hydrophobic polyNIPAAm phase. Probably, the slightly higher %W_r_ values for HG-9H result from the higher MM content. Thus, a similar effect was achieved with regard to enhancing the water absorption capacity with 0.52 mol% MM and 1.2 mol% NaAc. Calculating the amount of COOH, a higher molar content is incorporated with the MM, however, the acid resulting from the hydrolysis of EsterOxa, poly (2-carboxyethyl-2-oxazoline)s, has a pK_a_ of 5.6 [25], and thus, is weaker than that of other aliphatic carbonic acids with pK_a_ = 4.8 [26].

However, the MM containing hydrogels show a strong “aging effect”. After thorough drying and storage, a significant loss of the water absorption was noticed (see Table 2); for example, HG-9, which showed freshly prepared a Q value of 93, showed in reswelling experiments with 24 h conditioning in water only a Q value of 48. This effect indicates the formation of strong hydrogen bonding during the drying process, which cannot be broken completely when reswelling occurred for 24 h in water at pH 5.7. This phenomenon was not observed in the hydrogels with NaAc and thus, it is linked to the polyoxazoline MM structure, but may also relate to the fact that the pK_a_ value of poly (2-carboxyethyl-2-oxazoline)s is 5.6 [25], and thus, the acid is not fully dissociated at pH = 5.7. The lower Q values have to be considered in the following experiments, where the dried hydrogels are used in reswelling experiments.

#### 3.3.3. Temperature Sensitivity

**Figure 5:**
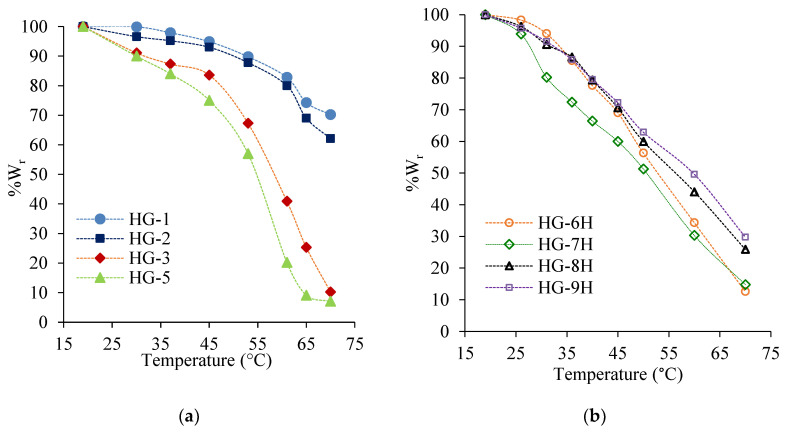
Temperature sensitivity for: (**a**) hydrogel HG-1 to -3, and -5 (**b**) hydrolyzed hydrogels HG-6H to -9H at pH 5.7.

As already demonstrated in the deswelling experiments, the hydrogels showed sensitivity to temperature due to the presence of polyNIPAAm segments in their structure. Figure 5a shows the temperature dependence of %W_r_ for NaAc-containing hydrogels HG-1 to HG-3 and for HG-5. It is obvious that the NIPAAm content in HG-1 and HG-2 is too low to result in a significant phase transition. Nevertheless, the content of absorbed water decreases by about 20–30% at 70 °C. The temperature effect is obvious for HG-3 with 58 mol% NIPAAm. Here, the curve becomes steeper at about 45 °C, and at 70 °C, the water is almost completely desorbed (%W_r_ ~ 10). The same is observed for HG-5 of comparable composition, but the curve is still even steeper and reaches %W_r_ ~ 10 at a temperature 5 K lower than HG-3. It is obvious that NaAc, which presents hydrophilic carboxylate groups, decreases the intensity of LCST of the polyNIPAAm segments [14]. Both DMAA as well as the small amount of NaAc, randomly distributed in the PNIPAAM gel structure (Scheme 3), lead to a significant shift of the volume phase transition temperature. Significant deswelling starts at about 45 °C, with a maximum of deswelling at ~ 55 °C (HG-5) and 60 °C (HG-3). A significant temperature dependency of %W_r_ is also observed for HG-6H to -9H (Figure 5b), similar as observed for HG-3 and HG-5. This is caused by the almost identical NIPAAm content (~60 mol%). However, even though the content of hydrolyzed MM varies, the effect of MM content is weak on the temperature-dependent deswelling. HG-6H to -9H deswell significantly between 30 and 70 °C, with those hydrogels with the highest MM content having still at 70 °C, a significant water content of 25 and 30%, respectively. However, compared to HG-3, a significant deswelling starts already at 35 °C, 10 °C lower, and temperature of maximum deswelling is at about 45 to 50 °C, also lower than for HG-3, even though the COOH content is higher. The conformational transition of the hydrogels due to the change in temperature was also evaluated by HR-MAS ^1^H NMR measurements on swollen samples. As demonstrated also for other NIPAAm-containing hydrogels [25,27], the intensities of the NIPAAm signals in the polymers are very sensitive to the hydrophilic–hydrophobic phase transition because the collapsed segments do not contribute to the narrow signal anymore.

**Figure 6:**
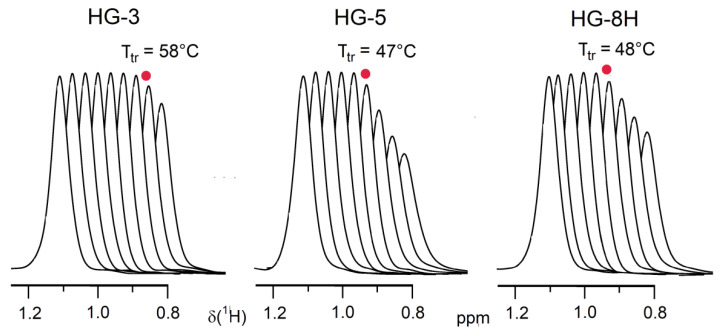
Stacked HR-MAS ^1^H NMR spectra (signal of the NIPAAm methyl group, all referenced on the chemical shift at 25 °C: 1.12 ppm) at different temperatures (25, 30, 35, 40, 45, 50, 55, 60 and 65 °C) for hydrogels HG-3, HG-5 and HG-8H swollen in D_2_O with transition temperature T_tr_.

Figure 6 depicts variable-temperature measurements of HG-3, HG-5 and HG-8H, with the intensity of the NIPAAm methyl group signal as the probe. The NMR measurements confirm the results from the water absorption measurements. The lowest transition temperature (T_tr_ = 47 °C) was determined for HG-5. Hydrophilic comonomers increase T_tr_, but this effect is more pronounced for NaAc units (T_tr_ = 58 °C for HG-3) in the backbone than for carboxylate groups in the MM side chain. For HG-8H, a T_tr_ = 48 °C was determined. This confirms that the effect of carboxylate groups in the MM side chain on T_tr_ is low, and thus, degree of swelling can be better adjusted with MM, resulting in a graft copolymer network structure (Scheme 3) without changing the PNIPAAm properties than with NaAc.

#### 3.3.4. Evaluation of the Mechanical Behavior and Thermal Stability of Hydrogels

**Figure 7:**
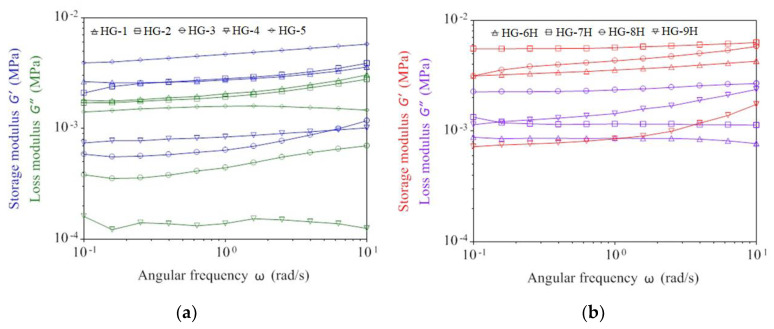
Frequency dependent measurements of G’ and G’’ of hydrogel series: (**a**) HG-1 to HG-5, (**b**) HG-6H to HG-9H at 22 °C.

Dynamic mechanical analysis allows verification of the elastic behavior of the synthesized materials. For that, the variation of storage modulus G’ (related to the capacity of the material to store energy) and loss modulus G’’ (related to the capacity of the material to dissipate energy) was evaluated at different values of angular frequency ω. For that measurement, dried hydrogels were conditioned for 24 h in water at 22 °C and pH = 5.7, meaning these are reswollen gels with lower Q values. Figure 7 shows the different graphs associated to each type of hydrogel and Table 3 and Table 4 summarize the moduli in relation to the Q values. For most hydrogel samples, the values of G’ and G” are nearly frequency-independent, and the values of G’ were greater than G’’ (with the exception of HG-9H). Therefore, in the following, the modulus values measured at an angular frequency of 10 rad/s are discussed. It can be concluded in analogy to Cipriano et al. [13] that the mechanical behavior for most of the synthesized polymeric materials corresponds to a behavior of a gel. Furthermore, due to the trend of G’ > G’’, a predominance of elastic behavior was confirmed. Evaluating more closely the results, one has to consider several aspects: firstly, the gels might not have reached their equilibrium degree of swelling or might swell inhomogeneously or have defects. This will lead to significant error in the measurements; thus, the determined absolute values should be looked at with caution and only trends should be discussed. Secondly, the elastic behavior of the gels is dominated by both the degree of crosslinking as well as by the degree of swelling. Both effects can be observed very nicely in the HG-1–HG-5 series (Figure 7a). The degree of crosslinking decreases from HG-1 to HG-4, which leads to a strong reduction in G’, especially for HG-3 and HG-4, with HG-4 not being stable anymore. At the same time, the Q value for HG-3 is double that of HG-2. Thus, the much lower G’ (1170 Pa) of HG-3 is easily understandable. The dominating effect of only the Q value can be seen when comparing HG-3 with HG-5, having similar crosslinking density but three times higher Q for HG-3. This results in a G’ of over 5700 Pa for HG-5, compared to 1170 Pa for HG-3.

Figure 7b compares the G’ values of the hydrolyzed graft hydrogels and Table 4 summarizes the G’ and G’’ values of both the MM hydrogels before and after hydrolysis. Since all those gels have similar DMAA values and thus, similar degree of crosslinking, the differences in the elastic behavior results from the degree of swelling, again, considering the above discussed caveat. Still, in general, one can state that the elastic module of the MM containing hydrogels are higher than those of the NaAc containing gels. G’ up to 9000 Pa was observed for non-hydrolyzed HG-9, and also HG-6H to HG-8H show G’ values up to 6000 Pa. This is on the one hand, the effect of the graft gel structures which proved in previous studies to lead to stiffer gels [18]; on the other hand, the above discussed strong hydrogen bonds which form during drying of the gels reinforce the gels, and subsequently, also lead to a lower Q value of only about 35. HG-9H, with a Q value of 48 in the reswollen state, shows also a very good stiffness as G’ is higher than 1700 Pa, which is significantly higher than that of HG-3. However, again, the higher degree of swelling leads to a lower G’.

**Figure 8:**
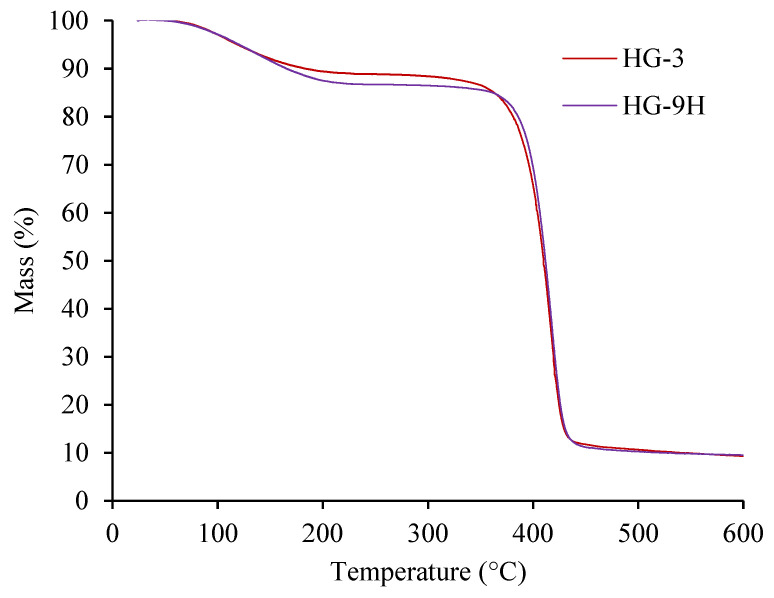
TGA curves of HG-3 and HG-9H (10 K/min under nitrogen atmosphere).

The thermal stability of some hydrogels was evaluated by thermogravimetric analysis (TGA) up to a temperature of 600 °C (Figure 8). From the obtained thermograms of the HG-3 and HG-9H hydrogels, it could be determined that these materials had thermal stability up to a temperature of approximately 350 °C. Up to 200 °C, the mass losses were around 10% by weight and could be associated with the water loss of the samples. Starting from 350 °C, the degradation of the hydrogels begins.

## 4. Conclusions

Self-crosslinked thermoresponsive hydrogels based on *N,N*-dimethylacrylamide and *N*-isopropylacrylamide have been successfully obtained. The composition has been optimized to achieve on the one hand, sufficient self-crosslinking through the DMAA monomers by redox initiation and to retain strong thermoresponsiveness due to the hydrophilic–hydrophobic phase transition of the NIPAAm components. A composition of about 40 mol% DMAA and 60 mol% NIPAAm was found to be optimal. In order to adjust the degree of swelling of the hydrogel, a small amount of carboxylic acid containing comonomers was added, specifically sodium acrylate (NaAc, < 1.66 mol%), randomly distributed in the DMAA/NIPAAm network. In addition, a styryl end functionalized polyoxazoline macromonomer (MM, COOH content < 2.6 mol%) was added for increasing hydrophilicity, which led to a graft copolymer hydrogel structure. MM was synthesized by ring-opening polymerization of 2-methyl-2-oxazoline (MeOxa) and methyl-3-(oxazol-2-yl)-propionate (EsterOxa) and about five carboxylic acid groups per MM chain were achieved after hydrolysis of the EsterOxa units. By that monomer combination with a small amount of COOH units, it was possible to achieve high Q (swelling) values of up to 90, with phase transition temperatures corroborated with deswelling of the hydrogels of 58 °C (HG-3, NaAc 1.2 mol% COOH) and 48 °C (HG-8H, MM, 1.75 mol% COOH), determined by T-dependent ^1^H NMR measurements. Thus, using the macromonomer approach, the phase transition caused by the PNIPAAm segments was less influenced by the polar comonomers due to the phase separation of the tangling MM chains in the graft copolymer hydrogel structure. Dynamic mechanical analysis verified the elastic behavior of the synthesized materials, which was dominated by both the crosslinking density given by the DMAA content and the degree of swelling. However, in general, higher moduli were obtained by the graft copolymer structures with the polyoxazoline tangling chains.

We can conclude that by optimizing the DMAA/NIPAAm ratio, it is possible to obtain efficiently self-crosslinked hydrogels with a strong temperature-sensitive phase transition. The water absorption capacity of those T-sensitive gels can be significantly increased by the addition of small amounts of COOH-containing comonomers (1–2.6 mol%), added as NaAc comonomer or as a COOH-containing polyoxazoline macromonomer, which led to a graft copolymer hydrogel. Compared to the gels having NaAc comonomers randomly distributed, the graft copolymer hydrogels provide additional advantages as there is a lower influence of the COOH-containing units on the phase transition temperature and higher storage modulus, thus a higher mechanical stiffness, especially of those gels with good swelling behavior. The resulting stiff and thermoresponsive hydrogels might have potential as actuator material with interesting system integration possibilities through the self-crosslinking feature. However, the present study shows the temperature sensitivity and mechanical properties on non-restraint hydrogels and further studies are needed to verify actuator capability e.g., in the restrains of a microfluidic setup.

## Figures and Tables

**Table 1:** Synthesis of hydrogels based on N,N-dimethylacrylamide, N-isopropylacrylamide and sodium acrylate ^a^ (HG-1–HG-5), Q values and experimental composition.

Hydrogel	DMAA ^a^(mol%)	NIPAAm ^a^(mol%)	NaAc ^a^(mol%)	Yield (%)	Q ^b^	DMAA_NMR_^c^ (mol%)	NIPAAm_NMR_^c^ (mol%)
HG-1	82.0	16.3	1.66	82	48 (31)	84	16
HG-2	59.9	38.9	1.1	81	44 (47)	62	38
HG-3	39.3	59.5	1.2	94	87 (83)	42	58
HG-4	19.1	79.9	1.0	91	- ^d^	24	76
HG-5	39.1	60.9	0	81	24 (28)	41	59

^a^ The feed composition is reported. The content of potassium persulfate (KPS) was 0.4 mol% of the total sum of monomers and the molar ratio TEMED/KPS was 1.3 in all experiments, ^b^ Water absorption degree at 20 °C and pH = 5.7; the values in brackets have been determined on freshly prepared swollen gels (W_s_), which were dried for the first time to obtain W_d_. In contrast to that, the regular Q values were obtained on strongly dried gels (in a drying oven at 40 °C and under vacuum) for determining W_d_ and after swelling for one day to obtain W_s_. ^c^ Molar content of DMAA and NIPAAM, respectively, in hydrogel determined from ^1^H NMR spectra (estimated error ± 2 mol%). The low content of NaAc could not be determined and was neglected in the calculations, ^d^ not determined due to the low mechanical stability of hydrogel.

**Table 2:** Synthesis of hydrogels based on *N,N*-dimethylacrylamide, *N*-isopropylacrylamide and 2-oxazoline macromonomer ^a^ (HG-6–HG-9), Q values and experimental composition.

Hydro-Gel	DMAA (mol%)	MM (mol%)	NIPAAm (mol%)	COOH ^b^ (mol%)	Yield (%)	Q ^c^HG HG-H		DMAA_NMR_^d^ (mol%)	NIPAAm_NMR_^d^ (mol%)	MM_NMR_^d,e^(mol%)
HG-6	39.54	0.09	60.38	0.45	71.1	25	33 (48)	38	62	0.08
HG-7	39.5	0.18	60.32	0.90	87.9	24	34 (50)	39	61	0.14
HG-8	38.57	0.35	61.05	1.75	85.0	21	34 (71)	38	62	0.25
HG-9	37.9	0.52	61.54	2.60	81.2	20	48 (93)	37	63	0.33

^a^ The feed composition is reported. The content of potassium persulfate (KPS) was 0.4 mol% of the total sum of monomers and the molar ratio TEMED/KPS was 1.3 in all experiments. ^b^ Molar amount of acid groups introduced with the MM after hydrolysis. ^c^ Water absorption degree at 20 °C and pH = 5.7; the values in brackets have been determined on freshly prepared swollen gels, which were dried for the first time to obtain W_d_. In contrast to the regular ones, Q values were obtained on strongly dried gels (in a drying oven at 40 °C) for determining W_d_ and after swelling 1 day to obtain W_s_. ^d^ Molar content of DMAA, NIPAAm and MM determined from ^1^H NMR spectra of the hydrolyzed hydrogels. ^e^ Calculated from MM backbone signal integrals considering DP = 24.

**Table 3:** Storage (G′) and loss (G″) moduli (at ω = 10 rad/s) of hydrogel series HG-1 to HG-5 at 22 °C.

Hydrogel	Q ^a^	G’ (Pa)	G’’ (Pa)
HG-1	48	3590	3045
HG-2	44	3880	2790
HG-3	87	1170	700
HG-4	-	1010	125
HG-5	24	5770	1465

^a^ Water absorption degree at 20 °C (pH = 5.7).

**Table 4:** Storage (G′) and loss (G’″) moduli (at ω = 10 rad/s) of hydrogel series HG-6 to HG-9 and HG-6H to HG-9H at 22 °C.

Hydrogel	Q ^a^	G’ (Pa)	G’’ (Pa)
HG-6	25	4220	1780
HG-7	24	2750	2010
HG-8	21	2880	3180
HG-9	20	9240	4710
HG-6H	33	4270	770
HG-7H	34	6300	1130
HG-8H	34	5810	2680
HG-9H	48	1740	2380

^a^ Water absorption degree at 20 °C (pH = 5.7).
